# Secretion Systems Used by Bacteria to Counteract Fungal Competitors

**DOI:** 10.3390/biology15090676

**Published:** 2026-04-25

**Authors:** Peishuai Fu, Liya Zhang, Xiaofang Ma, Xihui Shen, Lingfang Zhu, Changfu Li

**Affiliations:** State Key Laboratory for Crop Stress Resistance and High-Efficiency Production, Shaanxi Key Laboratory of Agricultural and Environmental Microbiology, College of Life Sciences, Northwest A&F University, Yangling 712100, China; fupeishuai2023@163.com (P.F.); zhang_liya1124@163.com (L.Z.); ma_xiaofang2026@163.com (X.M.); xihuishen@nwsuaf.edu.cn (X.S.); lingfangzhu@nwafu.edu.cn (L.Z.)

**Keywords:** bacterial secretion systems, bacterial–fungal interactions, antifungal mechanisms

## Abstract

Although bacterial secretion systems (BSSs) are well known to mediate antifungal effects, current evidence remains fragmented, and a framework linking antifungal strategies in bacterial–fungal warfare is still absent. In this context, we survey Type I to XI secretion systems, integrating experimentally validated activities with mechanism-based predictions of antifungal potential. On this basis, we highlight that the antifungal activity of BSSs operates through two different layers: direct fungicidal attack, via effector-mediated disruption of fungal cellular integrity, and ecological competition, through nutrient competition, physical niche occupation, and coordinated population-level activities. Nevertheless, the mechanistic understanding of bacterial antifungal strategies remains limited by simplified experimental models, incomplete effector repertoires, and poorly characterized regulatory networks and environmental cues. Addressing these gaps will require integrated experimental models, advanced high-throughput platforms, and comprehensive multi-omics-guided analyses to elucidate how bacteria suppress fungi and to support future applications. In addition, BSSs represent versatile and programmable platforms for the development of sustainable antifungal interventions in both agricultural and biomedical settings.

## Simple Summary

This review systematically summarizes the functions of type I-XI bacterial secretion systems in antifungal competition. We propose that bacterial antifungal strategies operate at two complementary levels: direct fungicidal attack, via effector-mediated disruption of cellular integrity, and ecological competition, through nutrient competition, physical niche occupation, and coordinated population-level activities. However, the current understanding of these mechanisms remains limited due to simplified experimental models, incomplete effector repertoires, and poorly characterized regulatory and environmental cues. Addressing this knowledge gap will require integrated experimental models, advanced high-throughput platforms, and comprehensive multi-omics analyses to unravel the mechanisms of bacterial fungal suppression. Moreover, bacterial secretion systems offer promising opportunities as programmable platforms for sustainable fungal control in agriculture and biomedicine.

## 1. Introduction

Bacteria and fungi frequently coexist in shared microhabitats, forming dynamic, coevolving communities [1]. As a result, they have established a remarkable array of sophisticated bacterial–fungal interactions (BFIs) [2], among which fierce competition for nutrients and ecological niches constitutes a central driving force [3]. This interkingdom competition is ubiquitous across diverse environments, from the human gut [4,5] to soil [6] and aquatic ecosystems [7], and profoundly shapes the structure and function of microbial communities [8,9]. To gain a competitive advantage in this perpetual conflict, bacteria employ a wide range of antagonistic mechanisms against fungi, including the production of secondary metabolites, siderophores, cell wall-degrading enzymes, and volatile organic compounds, as well as strategies involving nutrient competition and bacterial secretion systems (BSSs) [10,11,12].

BSSs are large protein complexes embedded in the cell membrane, functioning as sophisticated molecular delivery machineries that enable the transport of effectors either by direct injection into target cells or secretion into the extracellular environment [13,14]. These secreted proteins contribute to bacterial virulence through multiple mechanisms, including enhancing attachment to eukaryotic cells, acquiring nutrients in environmental niches, and directly targeting host cells to disrupt their physiological functions [15]. It has been widely reported that bacteria rely on specific secretion systems to mediate antifungal effects, highlighting their central role in interkingdom competition, such as the type II secretion system (T2SS) functioning as a chitinase transporter [16] and the type VI secretion system (T6SS) acting as a transkingdom weapon [17,18,19,20]. However, these findings have largely been reported in isolation, and a cohesive framework elucidating the overall strategy of bacterial antifungal defense remains undeveloped.

This theoretical gap further underscores the challenges involved in addressing the threats that fungi pose to human societies. In agriculture, the severity of fungal diseases affecting major crops has progressively increased since the early 20th century, resulting in estimated annual global yield losses of 10% to 23% [21,22]. In human health, fungi endanger well-being not only through direct infections but also via the toxic and allergenic effects of their metabolites [23]. Conventional antifungal approaches are increasingly constrained by the development of resistance and ecological concerns, prompting interest in alternative, biologically grounded strategies [24]. The urgent need for sustainable antifungal strategies highlights the importance of advancing our understanding of how bacteria deploy BSSs to counter fungal competitors.

This review systematically synthesizes the molecular mechanisms and functional diversity of type I to type XI secretion systems in bacterial antifungal strategies. Importantly, the available evidence for the involvement of different secretion systems in antifungal competition is not equally developed. Based on the current literature, their antifungal relevance can be broadly grouped into two evidence tiers, which are discussed in detail in the following sections. We further demonstrate that BSS-mediated antifungal activity can be organized into two functional layers: direct fungicidal attack and ecological competition. Finally, we evaluate current challenges in understanding bacterial antifungal mechanisms, propose potential solutions, and highlight the biotechnological potential of BSSs for fungal control in both agricultural and biomedical contexts.

## 2. Bacterial Secretion Systems (BSSs): Molecular Mechanisms and Antifungal Strategies

BSSs represent a diverse array of molecular machineries that enable bacteria to export enzymes, toxins, and other effectors involved in interspecies interactions [15]. In the context of antifungal competition, BSSs have been associated with diverse modes of action, ranging from direct interference with fungal cells to broader ecological effects. However, the strength of evidence varies considerably among different secretion systems. To provide a structured overview, the following sections examine type I to type XI secretion systems individually, focusing on their molecular characteristics, roles in antifungal competition, and the strength of current evidence. Based on the available literature, the antifungal relevance of these systems can be broadly divided into two evidence tiers: experimentally supported, referring to systems whose antifungal roles have been directly demonstrated through experiments, and putative, referring to systems whose antifungal relevance is inferred from mechanistic plausibility, characterized effectors, or ecological functions but have not yet been directly validated.

### 2.1. Type I Secretion System (T1SS)

The T1SS is a minimal transmembrane nanomachine in Gram-negative bacteria, consisting of only three major components: an intracellular ATP-binding cassette (ABC) transporter, a membrane fusion protein (MFP), and an outer membrane factor (OMF). Together, these components form a continuous channel that allows substrates to be transported directly from the cytoplasm across the inner and outer membranes [25,26,27].

In bacterial–fungal antagonism, the T1SS may mediate antifungal activity through two major mechanisms: competition for iron acquisition and secretion of antifungal enzymes [28,29]. First, the T1SS contributes to iron acquisition competition. Iron is an important micronutrient for both bacterial and fungal pathogens [30], serving as a redox-active cofactor in critical biological processes, including electron transport, iron–sulfur cluster biogenesis, and the regulation of virulence gene expression in diverse pathogens [31,32,33]. The T1SS secretes a protein named HasA, which binds heme and acquires iron from hemoglobin [28]. This function is postulated to enable competition with fungi for this vital resource, thereby potentially limiting iron availability to fungal competitors. Second, the T1SS may facilitate antifungal activity through the secretion of enzymes. Although no direct antifungal activity mediated by the T1SS has been demonstrated, lipases that can be secreted via this system [29] have been reported to disrupt fungal membrane integrity [34], suggesting a potential but unconfirmed role of the T1SS in antifungal interactions. Collectively, the available evidence suggests that the T1SS may represent a putative antifungal system, although this possibility still requires direct experimental validation.

### 2.2. Type II Secretion System (T2SS)

The T2SS, referred to as the secretion-dependent pathway, involves a two-step mechanism [35]. Initially, proteins are translocated through the inner membrane via either the Sec or Tat pathway. Subsequently, after a potentially brief interval in the periplasm, these proteins are transported from the periplasmic space to the extracellular environment by an outer membrane secretin [36,37,38].

The T2SS enables bacterial antifungal activity primarily through the secretion of chitinases, which degrade chitin in the fungal cell wall, and glucanases, which compromise glucans in the fungal cell wall [16,39,40]. In *Yersinia pseudotuberculosis*, sensing of fungal chitin activates the RstB-dependent T2SS/TadSS, resulting in the secretion of the antifungal effector TscE and direct antagonism toward fungal competitors [16]. Similarly, in *Burkholderia rhizoxinica*, the T2SS mediates direct antifungal activity through the secretion of chitinases that lyse fungal cells and enhance bacterial survival [39]. Additionally, the T2SS can secrete glucanases [40], including β-glucanases that degrade β-glucans in the fungal cell wall [41], thereby potentially contributing to bacterial antifungal activity. Taken together, these findings establish the T2SS as an experimentally supported extracellular antifungal system, with the strongest evidence centered on the secretion of chitin-targeting effectors.

### 2.3. Type III Secretion System (T3SS)

The T3SS constitutes a sophisticated nanomachine whose needle-like apparatus mediates the ordered secretion and translocation of effector proteins through the bacterial envelope and directly into target cells [42,43,44].

Based on this precise protein delivery mechanism, bacteria have adapted the T3SS to directly counteract fungal competitors by injecting effector proteins that disrupt vital cellular processes [45,46,47]. The T3SS effector ExoU possesses a cytotoxic function that targets fungal cells upon physical contact, as demonstrated in *Pseudomonas aeruginosa*, by functioning as a lipase that disrupts cellular membranes [45]. Moreover, the T3SS of *Burkholderia gladioli* NGJ1 secretes the effector Bg_9562, which directly mediates broad-spectrum antifungal activity against diverse fungi, including *C. albicans* and *Fusarium oxysporum*, leading to hyphal disintegration and growth inhibition, thereby enabling a mycophagous phenotype [46]. In addition, *Salmonella enterica* serovar Typhimurium employs its T3SS to deliver the effector SopB, an inositol phosphatase that suppresses *Candida albicans* hyphal growth and promotes bacterial competitive fitness in polymicrobial interactions [47]. Although these findings provide solid experimental evidence supporting that antifungal secretion systems enable bacteria to deliver effector proteins directly into fungal cells and disrupt vital cellular processes, current research evidence remains restricted to a small set of BFI models. It still remains unclear how widespread T3SS-mediated antifungal activity is among various bacterial taxa, and to what extent these interactions are governed by specific ecological environments. Additional research is required to clarify the generality and mechanistic diversity of T3SS-mediated antifungal activity.

### 2.4. Type IV Secretion System (T4SS)

The T4SS is a membrane-spanning, multi-subunit nanomachine that forms trans-envelope channels in Gram-positive bacteria, Gram-negative bacteria, and Archaea, enabling contact-dependent transfer of DNA or protein substrates [48,49,50].

The T4SS contributes to antifungal competition at both the single-cell and population levels, either directly or indirectly through gene transfer and protein-mediated interbacterial communication [51,52,53,54,55]. At the single-cell level, Peleg et al. proposed that the T4SS may mediate direct antifungal activity of *Acinetobacter baumannii* against *C. albicans* [51]. At the interpopulation level, the T4SS may be involved in the horizontal transfer of phenazine biosynthetic gene clusters, which encode antifungal metabolites, among bacterial populations, thereby potentially enhancing their collective antifungal competitive capacity [52,53,54]. In addition to this genetic dissemination, the T4SS has also been experimentally shown to mediate protein-based interbacterial communication that promotes antifungal activity. For instance, in *Lysobacter enzymogenes*, the T4SS enhances antifungal competitiveness by mediating the delivery of the effector LtaE into *Pseudomonas protegens*, where it activates 2,4-diacetylphloroglucinol (2,4-DAPG) production and thereby suppresses the fungal pathogen *Rhizoctonia solani* [55]. Thus, the T4SS should be regarded as experimentally supported in antifungal competition, primarily through protein-mediated interbacterial communication, whereas evidence for its direct single-cell antifungal activity and gene transfer-mediated ecological effects remains limited.

### 2.5. Type V Secretion System (T5SS)

The T5SS is the widespread protein secretion pathway in Gram-negative bacteria, characterized by an N-terminal signal peptide, a functional passenger domain, and a conserved C-terminal β-barrel translocator. The system is associated with diverse virulence-related functions, including adhesion, biofilm formation, proteolysis, and host invasion [56,57,58].

Current academic understanding provides no direct evidence for bacterial T5SS exerting fungicidal effects. Instead, this system may contribute to bacterial ecological advantage in fungal cohabitation environments through indirect strategies, including mediating bacterial adhesion and facilitating biofilm formation [58]. Nevertheless, the potential involvement of T5SS in BFIs cannot be excluded. Given that direct experimental evidence supporting its antifungal function remains absent and such biological roles are merely speculative at present, extensive research prospects still remain to be explored in this field.

### 2.6. Type VI Secretion System (T6SS)

The T6SS is a widespread Gram-negative secretion apparatus, consisting of a bacteriophage-like contractile sheath and a membrane-spanning complex, that injects protein effectors into target cells in a tightly regulated manner [59,60,61].

The T6SS serves as a highly effective antifungal armamentarium across diverse bacterial species, deploying effector proteins to inhibit or kill fungi through targeted delivery [17,18,19,20]. For instance, in *Serratia marcescens*, the T6SS is activated upon direct cell–cell contact to secrete effector proteins Tfe1 and Tfe2, which disrupt fungal membrane potential or nutrient uptake and amino acid metabolism, leading to cell death [17]. In addition, *A. baumannii* employs its T6SS to deliver the effector TafE, a DNase that kills fungi by degrading nuclear DNA within yeast cells [18]. Moreover, *Y. pseudotuberculosis* senses the fungal quorum-sensing molecule tyrosol via the EnvZ/OmpR system, thereby activating T6SS4 to deliver the antifungal effector TfeC and directly lyse *C. albicans* [19]. Furthermore, in agricultural environments, soil bacteria such as *Pseudomonas fluorescens* and *Lysobacter enzymogenes* employ the T6SS to directly inhibit pathogenic fungi through contact-dependent effector translocation, revealing an antibiotic-independent pathway for fungal suppression in agricultural ecosystems [20]. These findings establish the T6SS as an experimentally supported antifungal secretion system, with particularly strong evidence for direct fungicidal activity across diverse BFIs.

The T6SS is currently the most intensively studied and well-elucidated bacterial secretion system with antifungal functions, supported by abundant direct experimental evidence. However, several important issues remain to be addressed. Specifically, existing studies are mostly confined to a limited number of representative model systems. The regulatory mechanisms underlying T6SS activation and effector secretion in response to environmental factors, fungal-derived signals and interspecies contact cues across diverse habitats remain largely ambiguous. Meanwhile, the diversity of T6SS effectors determines the divergence of their antifungal mechanisms, which drives subsequent in-depth investigations.

### 2.7. Type VII Secretion System (T7SS)

The T7SS is a specialized protein export system predominantly found in mycobacteria and other Gram-positive bacteria, best known for its critical role in the virulence and intracellular survival of pathogens [62,63,64]. Homologous or functionally analogous systems have also been described in some Gram-negative bacteria [65], suggesting that T7SS-related secretion pathways may be more widespread than previously recognized.

While no direct experimental evidence links the T7SS to antifungal activity via effector secretion, its established role in metal homeostasis implies an indirect ecological impact. The T7SS, particularly the ESX-3 system, is essential for *Mycobacterium tuberculosis* to acquire iron and zinc, thereby maintaining metal-ion homeostasis [66]. Given that appropriate availability of these metals is indispensable for fungal growth, metabolism, and virulence [67], we hypothesize that T7SS-mediated metal acquisition in bacteria could influence fungal ecology by limiting resource availability in metal-poor environments. Thus, the possible contribution of the T7SS to antifungal competition should currently be regarded as putative and remains to be directly validated experimentally.

### 2.8. Type VIII Secretion System (T8SS)

The T8SS, also known as the curli biogenesis pathway, exports and assembles curli amyloid fibers on the bacterial surface [68]. These surface structures are fundamental for critical physiological processes, including bacterial adhesion, colonization of inert surfaces, and biofilm formation [69].

A systematic evaluation of the available evidence indicates that there is currently no direct experimental proof supporting an intrinsic antifungal function of the T8SS. Rather, this secretion system may contribute to bacterial ecological fitness in fungus-associated niches by promoting adhesion and facilitating biofilm development [69]. On this basis, the T8SS may likewise contribute to antifungal competition, but this possibility remains putative because direct experimental evidence is currently lacking.

### 2.9. Type IX Secretion System (T9SS)

The T9SS is a specialized multi-protein translocon exclusive to *Bacteroidetes*, which facilitates either gliding motility or virulence by recognizing a conserved C-terminal domain (CTD) on its cargo proteins, a domain that is cleaved during translocation and often modified with anionic LPS [70,71,72].

Current literature contains no direct reports of antifungal activity by effector molecules secreted via the T9SS. Although direct evidence is lacking, the T9SS is known to secrete a variety of enzymes, including glycoside hydrolases that degrade environmental polysaccharides [73,74]. For instance, *Flavobacterium johnsoniae* utilizes the T9SS to secrete chitinase, which degrades chitin, while *Chitinophaga pinensis* uses the T9SS to target both chitin and fungal β-glucans [73,74]. Together, these observations suggest a plausible mechanistic basis for antifungal activity and raise the possibility that the T9SS contributes to fungal antagonism, although this role has not yet been directly validated experimentally.

### 2.10. Type X Secretion System (T10SS)

The T10SS is a protein export pathway in Gram-negative bacteria that couples diverse holin membrane proteins with various cell wall-degrading enzymes to translocate hydrolytic enzymes and toxins from the periplasm to the extracellular space [75,76].

The direct role of the T10SS in antifungal attack remains to be fully substantiated by conclusive evidence. However, this system has been demonstrated to secrete chitinases in bacterial species such as *S. marcescens* and *S. enterica* serovar Typhimurium [76,77]. Since chitin constitutes an essential structural component of the fungal cell wall [78], this chitinase secretion capacity suggests a plausible mechanistic basis for T10SS-mediated antifungal activity, although direct experimental evidence for such a role is still lacking.

### 2.11. Type XI Secretion System (T11SS)

The T11SS is a DUF560-protein-based translocation apparatus in *Proteobacteria* that mediates dual substrate secretion: surface lipoprotein anchoring and soluble protein export, thereby facilitating bacterial environmental adaptation and host symbiosis [79,80].

Although no direct experimental evidence indicates that the T11SS mediates fungal inhibition or killing, it may indirectly affect fungal growth through nutrient acquisition. Grossman et al. summarized previous studies and concluded that homologous T11SS-dependent haem-binding proteins have been identified within the *Proteobacteria*, including HrpC from *Xenorhabdus nematophila*, HphA from *A. baumannii*, and Hpl from *Haemophilus haemolyticus* [80]. These haem-binding proteins enable certain bacteria to extract iron from heme [81], potentially conferring a competitive advantage over fungi in environments where iron is limiting. The above findings suggest that the T11SS may participate in BFIs indirectly via iron sequestration. Nevertheless, such a biological function remains merely speculative due to a lack of direct experimental evidence.

## 3. A Two-Layer Framework for BSS-Mediated Antifungal Competition

Competition is a key form of interaction between bacteria and fungi [8], and in ecology, it is broadly divided into two types: exploitative competition, which is indirect competition through resource consumption, and interference competition, which involves directly harming another organism [82]. BSSs have the potential to antagonize fungi by acting as weapons by delivering toxins and by facilitating resource acquisition through the secretion of siderophores or extracellular enzymes [12]. Based on their distinct modes of action and ecological strategies, we propose that BSSs employ two principal approaches to counter fungal competitors: direct fungicidal attack and ecological competition. Nonetheless, it is necessarily reductive, as natural BFIs are shaped by ecological context, community composition, and environmental signals beyond the scope of a static framework. Notably, we find that antifungal activity mediated by BSSs is not restricted to a single mode of action. Rather, certain secretion systems can employ both direct fungicidal attacks and ecological competition, highlighting the strategic flexibility of BSSs in responding to fungal competitors. At the same time, the secretion systems discussed here are supported by different levels of evidence: some have been directly implicated in antifungal activity through experimental studies, whereas others are included because their secreted effectors, ecological roles, or mechanistic properties suggest plausible but as yet untested contributions to fungal competition. Accordingly, this two-layer framework should be interpreted as an evidence-graded conceptual model that integrates both established antifungal mechanisms and hypothesis-generating functional predictions.

### 3.1. Direct Fungicidal Attack

Direct fungicidal attack involves effector-mediated disruption of fungal cellular integrity through the degradation of cell walls, disruption of membrane stability, or interference with critical intracellular processes. First, the T2SS delivers chitinases that hydrolyze chitin, a key structural component of the fungal cell wall, thereby weakening its integrity [16,39]. The T2SS has also been linked to glucanase secretion, suggesting a potential mechanism by which it could compromise fungal cell wall stability through targeting glucan polymers [40]. Second, the T3SS forms transient pores to inject effectors, including ExoU (a lipase that disrupts fungal cell membrane integrity), Bg_9562 (which mediates broad-spectrum antifungal activity, causing hyphal disintegration and growth inhibition) and SopB (an inositol phosphatase that suppresses fungal hyphal growth) [45,46,47]. Third, the T6SS utilizes contractile sheath propulsion to directly deliver effectors (including TafE, Tfe1, and TfeC) that target DNA, membrane potential, and fungal cell wall structures, ultimately causing irreversible cellular damage [17,18,19]. In addition, although direct evidence is lacking, the T1SS, T9SS, and T10SS also show potential antifungal activity: T1SS secretes lipases that could hydrolyze structural lipids in fungal cell membranes [29], while T9SS has been associated with the secretion of chitinases and glucanases, and T10SS with the delivery of chitinases, all of which could in principle degrade polysaccharide frameworks important for fungal structural stability [73,74,76,77]. Collectively, these systems illustrate a diverse arsenal that enables bacteria to execute sophisticated antifungal strategies.

### 3.2. Ecological Competition

The second strategy, ecological competition, constrains fungal proliferation through indirect environmental modulation, including nutrient sequestration, physical niche occupation, and coordinated population-level activities. These mechanisms limit fungal fitness and colonization through long-term ecological constraints rather than immediate fungicidal effects. Among the BSSs discussed here, experimental support is currently strongest for the T6SS and T4SS. In *S. marcescens*, the T6SS secretes the effector protein Tfe2, which interferes with fungal nutrient uptake and amino acid metabolism [17], providing direct evidence that this system can impair fungal fitness through metabolic interference. At the community level, the T4SS enhances antifungal competitiveness by enabling interbacterial effector delivery that stimulates antifungal antibiotic production in recipient cells, thereby coordinating population-level antifungal responses [55], although its contribution through horizontal transfer of antifungal gene clusters remains less well established [52,53,54]. By contrast, the involvement of other secretion systems in ecological competition remains largely putative. For example, the T1SS secretes HasA, which sequesters iron [28], potentially influencing fungal growth indirectly in shared environments. Likewise, the T7SS and T11SS may be relevant to nutrient competition through their associations with metal ion homeostasis and hemophore-mediated iron acquisition [66,80]. In addition, the T5SS and T8SS may contribute to spatial dominance by mediating surface adhesion and biofilm assembly, which could reduce the availability of colonization sites for fungi [56,69]. These systems form a multilayered ecological competition strategy that reshapes local microenvironments through niche occupation, nutrient competition, and coordinated population-level regulation, imposing sustained constraints on fungal competitors.

## 4. Future Directions

Although antifungal roles have been clearly established for a subset of BSSs [12], several important obstacles continue to hinder a comprehensive understanding of bacterial antifungal mechanisms. First, the inherent complexity of BFIs is poorly captured by current experimental models, which fail to replicate the multi-species networks and dynamic environmental fluctuations found in natural ecosystems [83,84]. Second, the effector repertoires of BSSs remain far from complete, as exemplified by previous reports on the T6SS, for which a large number of effector proteins remain unidentified [85]. Third, while it is known that bacteria perceive fungal cues either through transmembrane signaling systems or via physical contact, enabling the precise activation of specialized secretion systems that deliver antifungal effectors [1,12], the limited number of studied cases has constrained our understanding of the regulatory networks and environmental cues controlling BSS activation in BFIs. This limits our ability to determine when and under what conditions these systems are functionally engaged. Collectively, these factors substantially hinder the elucidation of BSS-mediated fungal interactions.

To systematically address the aforementioned challenges, future research should establish an integrated technological framework. First, high-throughput platforms such as microfluidic chips could be employed to construct synthetic microbial communities [86,87,88], and it is also possible to integrate molecular mechanism research with dynamic environmental simulation by introducing fluctuations in environmental parameters such as pH, temperature, and nutrient availability within controlled systems [89], enabling the analysis of the function of BSSs within simulated complex natural environments. In addition, bioinformatic prediction coupled with systematic identification tools, including CRISPR-Cas9 screening, and mass spectrometry (MS) imaging [90,91,92,93], may provide valuable guidance for identifying BSS effector proteins and characterizing their expression and spatial distribution. Moreover, integrative multi-omics strategies, particularly metabolomics combined with transcriptomic and proteomic analyses [94], could offer a conceptual and technical foundation for future efforts aimed at predicting and dissecting the regulatory networks and environmental cues governing BSS activation during BFIs. Together, these approaches provide a foundational framework to advance our understanding of bacterial antifungal mechanisms in ecologically relevant contexts, which links molecular processes with environmental complexity.

The challenges posed by fungi and the harm they induce are both persistent and severe for human societies [95,96,97], raising the question of whether the efficient “cargo delivery” capability of BSSs can be harnessed for antifungal control in agricultural and biomedical contexts. Notably, BSSs have been shown to have great potential in biotechnological and biomedical applications for their exceptional substrate delivery capacity [98]. This is exemplified by the T1SS, which efficiently secretes diverse heterologous proteins, including recombinant products and small polypeptides [99,100]. Similarly, T3SS enables direct translocation of therapeutic cargo, such as antigens, nucleases, and transcription factors, into mammalian cells [101,102]. These findings collectively suggest the versatility and certain applicability of BSSs in the production and delivery of heterologous proteins.

Building on this foundation, we argue that BSSs could provide a flexible and engineerable framework for antifungal control, supporting diverse intervention strategies ranging from virulence factor neutralization and fungicidal structural damage to ecological competition. Inspired by T1SS-based secretion of antibody fragments [99,100], engineered bacteria can be designed to secrete nanobodies or resistance proteins that specifically bind and neutralize key virulence factors of fungal pathogens. In addition, antifungal strategies can be achieved by directly targeting essential structural components of fungal cells, with secretion systems such as T1SS, T3SS, and T6SS being repurposed to deliver antifungal effectors, including hydrolytic enzymes and engineered toxins, thereby directly compromising essential cellular structures. Beyond direct molecular targeting, BSSs may be valuable in antifungal applications that rely on ecological engineering rather than direct fungicidal attack. In this context, secretion-associated functions such as surface colonization, biofilm formation, spatial exclusion, and resource capture could be strategically exploited. Together, these functions may help build microbial communities that are more resistant to fungal establishment and expansion. These properties may be particularly useful in agricultural and host-associated settings, where long-term suppression of fungal invasion often depends not only on toxicity, but also on sustained niche protection and community stability [11,103]. In synthetic or engineered microbiomes, secretion-linked traits could therefore be leveraged to improve bacterial persistence, reinforce cooperative interactions, and enhance occupation of vulnerable niches before fungal pathogens become established. More broadly, this ecological mode of intervention expands the application potential of BSSs beyond effector delivery alone, positioning them as tools for shaping microbiome structure and function in ways that favor antifungal resilience.

Nevertheless, several important challenges must be addressed before translating BSSs into reliable bioengineering and antifungal applications. The environmental fitness of engineered bacteria remains poorly defined under variable field or host conditions, restricting their functional stability and persistence in complex ecosystems. Serious biosafety issues also require careful assessment prior to practical use, including the risk of horizontal gene transfer, ecological disturbance, and unintended impacts on non-target microbes. In addition, effector targeting and delivery often lack sufficient specificity, raising the potential for off-target activity and poor controllability in natural settings. Engineered BSS modules also suffer from limited stability within complex microbial communities, as interspecies interactions and environmental stress often compromise designed functions. Collectively, these constraints call for optimized design strategies that integrate anti-microbial efficacy, target specificity, biosafety, and ecological compatibility. Future efforts should prioritize the development of robust, context-dependent regulatory circuits, precision-targeted delivery systems, and minimized fitness burdens to enable safe and sustainable application of BSS-based strategies in agriculture, biomedicine, and microbiome engineering.

## 5. Conclusions

In conclusion, this review provides a systematic synthesis and comparative analysis of type I-XI BSSs in antifungal competition, as summarized in Table 1. Based on the evidence summarized above, we argue that bacterial antifungal strategies operate at two complementary levels, as summarized in Table 2: direct fungicidal attack via effector-mediated disruption of fungal cellular integrity, and ecological competition through nutrient competition, physical niche occupation, and coordinated population-level activities. Because only a subset of secretion systems has been directly linked to antifungal activity, this framework should be regarded as an evidence-graded model rather than a definitive classification. In addition, mechanistic understanding remains limited by simplified experimental models, incomplete effector repertoires, and poorly characterized regulatory and environmental cues. Addressing these gaps will require integrated experimental models, advanced high-throughput platforms, and comprehensive multi-omics analyses to fully elucidate BSS-mediated antifungal mechanisms. Moreover, this review highlights the potential of BSSs as programmable platforms for sustainable antifungal applications in agricultural and biomedical contexts.

## Figures and Tables

**Table 1:** BSSs: molecular mechanisms and antifungal strategies.

Secretion System Type	Major Bacterial Hosts	Core Molecular Mechanism Brief Introduction	Antifungal Mechanisms and Effectors	Antifungal Strategy	Evidence Category	References
Type I (T1SS)	Gram-negative bacteria	Continuous channel of ABC, MFP, and OMF for transport.	Lipase, HasA	Direct Fungicidal Attack; Ecological Competition	Putative	[28,29]
Type II (T2SS)	Gram-negative bacteria	Two-step: Sec/Tat to periplasm, then outer membrane secretin.	Chitinases, glucanases	Direct Fungicidal Attack	Experimentally supported	[16,39,40]
Type III (T3SS)	Gram-negative bacteria	Needle-like injector for direct effector delivery into target cells.	Bg_9562, ExoU, SopB	Direct Fungicidal Attack	Experimentally supported	[45,46,47]
Type IV (T4SS)	Gram-negative, Gram-positive, Archaea	Contact-dependent transfer of DNA or proteins.	DNA (gene clusters), Protein effectors	Ecological Competition	Experimentally supported	[51,52,53,54,55]
Type V (T5SS)	Gram-negative bacteria	Sec-dependent periplasmic transfer and β-barrel-mediated export of the passenger domain.	bacterial adhesion, biofilm formation	Ecological Competition	Putative	[56,57,58]
Type VI (T6SS)	Gram-negative bacteria	Contractile, phage-like sheath injects effectors.	Tfe1, Tfe2, TafE, TfeC, Le1893	Direct Fungicidal Attack; Ecological Competition	Experimentally supported	[17,18,19,20]
Type VII (T7SS)	Primarily Gram-positive	Translocation of proteins across the bacterial cell envelope.	Proteins for metal homeostasis	Ecological Competition	Putative	[66]
Type VIII (T8SS)	Gram-negative bacteria	Curli biogenesis pathway; exports amyloid fibers.	Mediating adhesion and facilitating biofilm formation	Ecological Competition	Putative	[69]
Type IX (T9SS)	Specific Phylum: *Bacteroidetes*	CTD-recognizing translocon	Glycoside Hydrolases	Direct Fungicidal Attack	Putative	[73,74]
Type X (T10SS)	Gram-negative bacteria	Holins coupled with hydrolases for enzyme export.	Chitinase	Direct Fungicidal Attack	Putative	[76,77]
Type XI (T11SS)	Specific Phylum: *Proteobacteria*	DUF560-based apparatus for protein export/anchoring.	Heme-binding proteins	Ecological Competition	Putative	[80]

**Table 2:** Two Mechanistic Layers of Antifungal Activity in Bacterial Secretion Systems.

Functional Paradigm	Core Strategic Principle	Involved Secretion Systems	Evidence Category	Representative Mechanisms
Direct Fungicidal Attack	Direct fungicidal attack involves effector-mediated attacks that compromise fungal cellular integrity by degrading cell walls, disrupting membrane stability, or interfering with critical intracellular processes.	T1SS, T2SS, T3SS, T6SS, T9SS, T10SS	Experimentally supported: T2SS, T3SS, T6SS; Putative: T1SS, T9SS, T10SS	Cell wall damage; membrane disruption; intracellular damage.
Ecological Competition	Ecological competition constrains fungal proliferation through indirect environmental modulation, including nutrient competition, physical niche occupation, and coordinated population-level activities.	T1SS, T4SS, T5SS, T6SS, T7SS, T8SS, T11SS	Experimentally supported: T4SS, T6SS; Putative: T1SS, T5SS, T7SS, T8SS, T11SS	Nutrient competition; spatial exclusion; population-level coordination; biofilm-associated persistence.

## Data Availability

No new data were created or analyzed in this study. Data sharing is not applicable to this article.

## Abbreviations

The following abbreviations are used in this manuscript:

BSSs	Bacterial secretion systems
BFIs	Bacterial–fungal interactions
T1SS	Type I secretion system
T2SS	Type II secretion system
T3SS	Type III secretion system
T4SS	Type IV secretion system
T5SS	Type V secretion system
T6SS	Type VI secretion system
T7SS	Type VII secretion system
T8SS	Type VIII secretion system
T9SS	Type IX secretion system
T10SS	Type X secretion system
T11SS	Type XI secretion system
ABC	ATP-binding cassette
MFP	Membrane fusion protein
OMF	Outer membrane factor
Sec	Secretory pathway
Tat	Twin-arginine translocation
TadSS	Tad secretion system
2,4-DAPG	2,4-diacetylphloroglucinol
ESX-3	Type VII secretion system subtype 3
CTD	C-terminal domain
LPS	Lipopolysaccharide
DUF560	Domain of unknown function 560
CRISPR-Cas9	Clustered regularly interspaced short palindromic repeats-CRISPR-associated protein 9
MS	Mass spectrometry
